# Regulation of Dendrobium Polysaccharides on Proliferation and Oxidative Stress of Human Umbilical Vein Endothelial Cells in the High Glucose Environment

**DOI:** 10.1155/2021/6685055

**Published:** 2021-06-13

**Authors:** Yajia Li, Ziqin Cao, Limin Jia, Yanfei Huang, Meilan Shi, Qiangxiang Li

**Affiliations:** ^1^Department of Dermatology, Xiangya Hospital, Central South University, Changsha, China; ^2^National Clinical Research Center for Geriatric Disorders, Xiangya Hospital, Central South University, Changsha, Hunan, China; ^3^Ningxia Geriatric Disease Clinical Research Center, People's Hospital of Ningxia Hui Autonomous Region, Yinchuan, Ningxia Hui Autonomous Region, China; ^4^Department of Spine Surgery, The Xiangya Hospital, Central South University, Changsha, Hunan, China; ^5^Loudi Central Hospital, the Loudi Affiliated Hospital of Nanhua University, Hunan province, Loudi, 417000, China; ^6^Hunan People's Hospital, Department of Hunan Institute of Geriatrics, Changsha, China

## Abstract

**Backgrounds:**

Polysaccharides of Dendrobium candidum (PDC) showed a strong antioxidant effect on islet cells while the effects of PDC on human umbilical vein endothelial cells (HUVECs) under the high glucose condition remain unclear. *Material and Method*. HUVECs were incubated with high glucose (33.3 mmol/L) for 48 hours to induce injury, and cells were treated with PDC (100, 200, and 400 *μ*g/mL) for 48 hours. The tetrazolium blue colorimetric (MTT) assay was used to detect cell proliferation, superoxide dismutase (SOD), and nitric oxide (NO) content in cell supernatants. Flow cytometry was used to detect reactive oxygen species (ROS) and calcium levels.

**Results:**

(1) Compared with the control group, the proliferation of HUVECs cells in the high glucose (33.3 mmol/L) group decreased (*P* < 0.05). The intracellular calcium ion concentration and the intracellular ROS level increased (*P* < 0.01 and *P* < 0.05). SOD activity and the level of NO in the culture medium were reduced (*P* <0.05). (2) Compared with the control group, PDC (50, 100, 200, 400, and 800 *μ*g/mL) did not significantly affect the cell proliferation of HUVECs (*P* > 0.05). (3) Compared with the high glucose group, the HUVEC cell viability of the high glucose + PDC (100, 200, and 400 *μ*g/mL) group increased while the intracellular calcium ion concentration decreased in a concentration-dependent manner (*P* < 0.05). Intracellular ROS levels were reduced, while SOD activity and the level of NO in culture fluids increased (*P* < 0.05).

**Conclusion:**

PDC can promote the proliferation of HUVECs in the high glucose environment by reducing oxidative stress injury of HUVECs induced by high glucose.

## 1. Background

The major chronic complications of diabetes (diabetes mellitus, DM) involve the diseases of heart, eye, kidney, foot, and neuropathy, all related to vascular injury [1, 2]. Many researchers have focused on diabetic vascular disease but the pathogenesis of diabetic vascular disease has yet to be further clarified. Increased oxidative stress is one of the pathogenesis of diabetic microvascular complications. Its role in the chronic pathogenesis of diabetes is not only direct cytotoxic damage but also can act as an important intracellular messenger to activate signaling pathways, directly leading to tissue and cell damage [3, 4]. Previous studies have confirmed that increased reactive oxygen species (ROS) production can lead to decreased endothelial cell survival, and the induction of endothelial cell damage is related to increased intracellular free calcium and activation of ROS [1, 5, 6]. Intracellular levels of reactive ROS and free calcium can contribute to dysfunction and progressive loss of beta cells and thereby to diabetes mellitus. Besides, as an antioxidant enzyme in mitochondria, superoxide dismutase (SOD) plays an important role in combating oxidative damage. The decrease of SOD activity is an important biochemical characteristic of diabetic patients. Decreased endothelium-dependent vasodilation in diabetes may be related to both the reduced nitric oxide (NO) synthesis and increased NO inactivation [7, 8]. Dendrobium polysaccharide, also called polysaccharides of Dendrobium candidum (PDC), is the main active ingredient in Dendrobium candidum [9]. Studies have shown that PDC has a strong antioxidant effect in vitro, which can inhibit islet cell apoptosis and necrosis, protect islet cells, resist calcium overload, and protect and repair cells [10–12].

Therefore, we speculated that PDC might regulate the oxidative stress of human umbilical vein endothelial cells (HUVECs) under high glucose environment, promote the proliferation of HUVECs, and thus exert its effect on protecting endothelial cells. This study was to explore the effects of PDC on HUVECs by detecting the SOD activity and NO content, as well as the ROS and calcium levels in high glucose conditions.

## 2. Materials and Methods

### 2.1. Experimental Materials

HUVECs were purchased from the Institute of Pharmacology of Central South University; active oxygen detection kit, Fluo-3AM (calcium ion fluorescence probe), was purchased from (Jiangsu Province Biyuntian Institute of Biotechnology); PDC was prepared by this research group; low glucose DMEM culture solution was purchased from (Gibco, USA); fetal bovine serum was purchased from (APP, Australia); D-glucose and trypsin powder was purchased from (Sigma, USA); CO2 culture box was purchased from (Shelodn company); inverted biological microscope was purchased from (Japan OLYMPUS company); automatic microplate reader was purchased from (Japan CORONA company); LDZX-40C vertical self-control electric heating pressure steam sterilizer was purchased from (Shanghai Shenan Medical Instrument Factory); KH -600DB data ultrasonic cleaner was purchased from (Kunshan Hechuang Ultrasonic Instrument Co., Ltd.); flow cytometer was purchased from (BECTON DICKINSON Company, USA).

### 2.2. Establishment of the High Glucose Model and Experimental Grouping after Cell Culture and Generation of Umbilical Vein Endothelial Cells

When the cells reach 80% confluence, discard the serum-containing culture medium, change the serum-free medium for 24 hours (h), and then change to a culture medium with different concentrations of glucose, which are 17.5 mmol/L and 25 mmol/L, and 33.3 mmol/L, 40 mmol/L, and 56 mmol/L. After observation at 12, 24, 36, 48, and 72 h, the optimal glucose concentration for establishing a high glucose model of umbilical vein endothelial cells was 33 mmol/L.

### 2.3. Experimental Grouping

#### 2.3.1. Effect of Different Concentrations of PDC on the Growth of HUVECs

The study was done on HUVECs treated for 48 hours in different groups: one control group and other groups with different concentrations of PDC (50, 100, 200, 400, and 800 *μ*g/mL).

#### 2.3.2. Effects of Various Glucose Concentrations on the Proliferation of HUVECs

The study was done on HUVECs treated with glucose for 48 hours in different groups: one control group and other groups with different concentrations of glucose (17.5 mmol/L, 25 mmol/L, 33.3 mmol/L, 40 mmol/L, and 56 mmol/L).

#### 2.3.3. Effect of PDC on the Growth of HUVECs under High Glucose Environment In Vitro

The study was done on cell treated for 48 hours in groups with high concentration like high glucose group (33.3 mmol/L) and high glucose + different concentrations of PDC (100, 200, and 400 *μ*g/mL) compared to the control group.

#### 2.3.4. Effect of PDC on HUVEC Active Oxygen and Intracellular Calcium Ion Concentration, Superoxide Dismutase Activity, and Nitric Oxide Content in Cell Supernatant under the High Glucose Environment In Vitro

The experiment was divided into a control group, a high glucose group (33.3 mmol/L), and a high glucose + PDC (100, 200, and 400 *μ*g/mL) cotreatment group. The treatment time was 48 hours. Set five replicates in each group and repeated three times.

### 2.4. Tetrazolium Blue Colorimetric (MTT) Method Was Used to Detect Cell SOD and Nitric Oxide (NO) Content

HUVEC was washed with PBS and digested, and cell suspension was made with the M200 medium containing 0.5% FBS. After counting the cells, seed is 5 × 10^3^/150 *μ*L per well in a 96-well plate. The whole medium was cultured for 24 hours, the culture medium was discarded, and the medium was only pretreated with 0.5% FBS for 24 hours to achieve cell synchronization. Add the control group, experimental group, and positive control medium. After continuing the normal culture for 12, 24, 36, 48, and 72 hours, 50 *μ*L of MTT solution was added, and the cell proliferation was measured by the microplate reader using the MTT method at 490 nm and 690 nm (OD) of each well. Set 3 replicates in each group and repeat the experiment at least three times. Measurement of NO: set up a blank tube (no sample and sodium nitrite added), a standard tube (add 20 *μ*mol/L sodium nitrite), and a determination tube (add 0.3 mL of the supernatant to be tested). The tubes were mixed and placed for 10 minutes, centrifuged at 3500~4000 r/min for 10 minutes, and 0.8 mL of the supernatant was taken. Add 0.4 mL of developer and mix well and measure the absorbance after 15 minutes. Find the corresponding NO concentration in the standard curve (sodium nitrite series solution) prepared in advance. Absorbance of NO was measured at 540 nm; measurement of SOD: set up a test tube and a control tube according to the requirements of the test box and add various reaction solutions and the supernatant to be tested to the test tube and mix well. Set the temperature constantly of the water bath to 37°C for 40 min, add the color reagent and mix well, pour it into a 1 cm optical path cuvette after 10 min, adjust to zero with distilled water, and compare the color with a microplate reader at a wave length of 550 nm. According to the formula SOD activity (U/mgprot) = [(control tube − measurement tube)/control tube] ÷ 50% × (total volume of reaction solution/sampling amount) ÷ protein content in tissue (mgprot/mL), to calculate SOD activity, no clear sample is added to the control tube, and the others are the same as the measurement tube [13–15].

### 2.5. Detection of ROS and Calcium Ion by Flow Cytometry

The cells over 75 ml bottles were evenly seeded on a six-well plate, and PDC (100, 200, 400 *μ*g/ml) was added for 48 h. Each group was set up with 5 duplicate wells and repeated three times; the culture solution was aspirated. Each group contains three parallel samples. Detection and analysis by flow cytometry. The recommended excitation wavelength is 488 nm, and the emission wavelength is 525-530 nm. Reactive Oxygen Species Assay Kit is one of the most commonly used methods to quantitatively detect the level of reactive oxygen species in cells by the change in fluorescence intensity of the fluorescent dye DCFH-DA. Fluo-3 AM is a fluorescent dye that penetrates cell membranes. Fluo-3 can be combined with calcium ions, which can produce strong fluorescence when combined with calcium ions. The maximum excitation wavelength is 506 nm, and the maximum emission wavelength is 526 nm.

### 2.6. Data Statistics and Analysis

All data in the experiment are expressed as mean ± standard deviation ($\bar{x}\pm \text{s}$), and statistical processing is performed using international general statistical software SPSS16.0. The experimental data were analyzed by one-way ANOVA, and the pairwise comparison of different groups was performed by the SNK-q test. *P* < 0.05 was considered significant.

## 3. Results

### 3.1. Effect of PDC or High Glucose In Vitro on HUVEC Proliferation

As shown in Table 1, according to the results of MTT, compared with the control group, PDC at 50, 100, 200, 400, and 800 *μ*g/mL did not significantly inhibit HUVECs (*P* > 0.05). Therefore, it can be judged that PDC has no obvious cytotoxic effect. Further, explore the effects of different glucose concentrations on the proliferation of HUVECs, as shown in Table 2: compared with the control group (OD value was 1.09 ± 0.14), the concentration of 25.5, 33.3, 40.0, and 56.0 mmol/L glucose significantly inhibited HUVEC cell proliferation with OD values of 0.75 ± 0.09, 0.42 ± 0.05, 0.31 ± 0.04, and 0.19 ± 0.03, respectively, which also showed a concentration dependence (*P* < 0.05). When the concentration reached 33.3 mmol/L, the inhibition rate of HUVEC cell proliferation was 51.65%, which was the optimal glucose concentration in the culture medium. However, the cell proliferation ability of the mannitol (56.0 mmol/L) group was not significantly different from that of the control group (*P* > 0.05), indicating that high glucose caused reduced HUVEC cell proliferation, and induced cell damage was not related to osmotic pressure. The results are shown in Table 3: compared with the high glucose group (OD value was 0.43 ± 0.05), the cell proliferation of the high glucose + PDC (100, 200, and 400 *μ*g/mL) group significantly increased (OD values were 0.58 ± 0.06, 0.78 ± 0.08, and 0.92 ± 0.08, respectively), showing a concentration dependence (*P* < 0.05). The results show that PDC has a protective effect on protecting HUVECs from high glucose.

### 3.2. Effect of PDC on Intracellular Calcium Concentration of HUVEC Cells Cultured in High Glucose In Vitro

**Figure 1:**
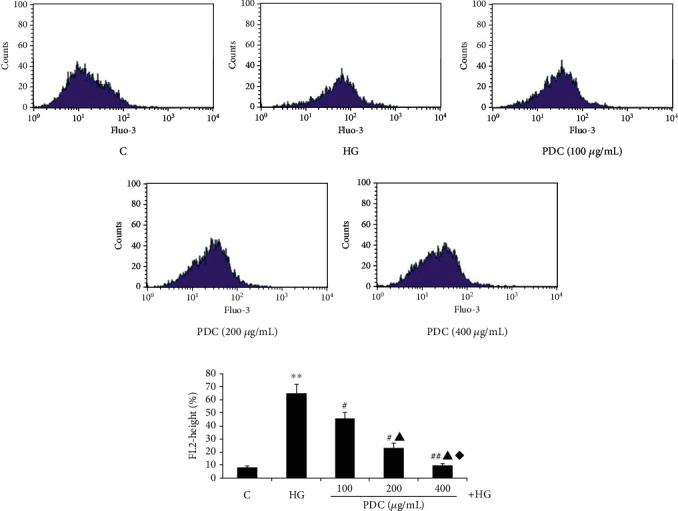
(a, b) Effect of PDC on calcium in HUVEC cells cultured in high glucose. Both D-glucose (33.3 mmol/L) and PDC (100, 200, and 400 *μ*g/mL) were incubated with HUVECs for 48 hours. Fluo-3 AM is a fluorescent probe for detecting calcium in cells by flow cytometry. C: control group; HG: high D-glucose group; PDC: Dendrobium candidum polysaccharide group. All data are expressed as $\bar{𝓍}\pm \text{S }$ (*n* = 5). ^∗∗^*P* < 0.05, compared with the control group; #*P* < 0.05, ##*P* < 0.01 compared with the high glucose group; ▲*P* < 0.05, compared with the high glucose + PDC (100 *μ*g/mL) group; ◆*P* < 0.05, compared with high glucose + PDC (200 *μ*g/mL) group.

The results are shown in Figures 1(a) and 1(b). Compared with the control group, the intracellular calcium fluorescence intensity of HUVECs in the high glucose group significantly increased (the intracellular calcium ion fluorescence intensity was 12.15 ± 1.78 and 62.46 ± 5.86, respectively, *P* < 0.01); compared with the high glucose group, the high glucose + PDC (100, 200 and 400 *μ*g/mL) group significantly reduced the fluorescence intensity of calcium ions, in a concentration-dependent manner (intracellular calcium ion fluorescence intensity was 42.20 ± 5.76, 26.28 ± 3.11, and 11.49 ± 2.48, *P* < 0.05). These indicate that PDC can prevent intracellular calcium overload caused by high glucose.

### 3.3. Effect of PDC on Reactive Oxygen Species and ROS in HUVEC Cells Cultured in High Glucose In Vitro

**Figure 2:**
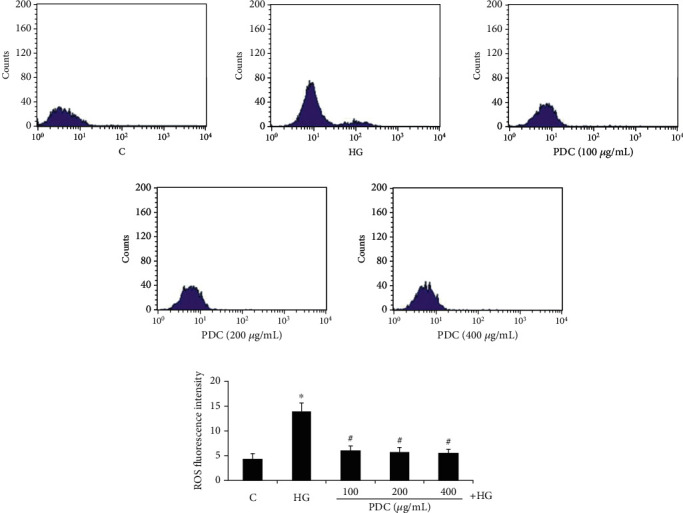
(a, b) Effect of PDC on ROS levels in HUVECs cultured in high glucose. Both D-glucose (33.3 mmol/L) and PDC (100, 200, and 400 *μ*g/mL) were incubated with HUVECs for 48 hours. After staining with the fluorescent dye DCFH-DA, the intracellular ROS level was detected by flow cytometry. C: control group; HG: high D-glucose group; PDC: Dendrobium candidum polysaccharide group. All data are expressed as $\bar{𝓍}\pm S$ (*n* = 5). ^∗^*P* < 0.05, compared with control group; #*P* < 0.05, compared with high glucose group.

The results of flow cytometry were shown in Figures 2(a) and 2(b). Compared with the control group, the generation of reactive oxygen species in HUVEC cells in the high glucose group significantly increased (ROS fluorescence intensity was 4.41 ± 0.96 and 14.00 ± 1.64, respectively, *P* <0.05); compared with the high glucose group, the levels of reactive oxygen species in the high glucose + PDC (100, 200, and 400 *μ*g/mL) group significantly reduced (ROS fluorescence intensity was 6.14 ± 0.83, 5.83 ± 0.86, and 5.61 ± 0.72, respectively, *P* < 0.05).

### 3.4. Effect of PDC on SOD in HUVEC Cells Cultured in High Glucose

**Figure 3:**
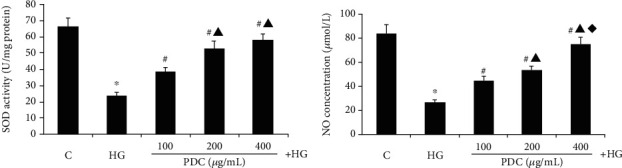
(a) Effect of PDC on SOD activity in HUVECs cells cultured in high glucose. (b) Effect of PDC on NO secretion from HUVECs cells cultured in high glucose. Both D-glucose (33.3 mmol/L) and PDC (100, 200, and 400 *μ*g/mL) were incubated with HUVECs for 48 hours. Detection of SOD activity and NO secretion. C: control group; HG: high D-glucose group; PDC: Dendrobium candidum polysaccharide group. All data are expressed as $\bar{x\pm S}$ (*n* = 5). ^∗^*P* < 0.05, compared with the control group; #*P* < 0.05, compared with the high glucose group; ▲*P* < 0.05, compared with the high glucose + PDC (100 *μ*g/mL) group; ◆*P* < 0.05, compared with the high glucose + PDC (200 *μ*g/mL) group.

As shown in Figure 3(a), compared with the control group, the intracellular SOD activity of the high glucose group significantly reduced (SOD activities were 66.14 ± 1.70 and 24.02 ± 1.67 U/mg protein, respectively, *P* < 0.05). Compared with the high glucose group, the intracellular SOD activity in the high glucose + PDC (100, 200, and 400 *μ*g/mL) group significantly increased (SOD activities were 38.92 ± 2.08, 52.98 ± 2.23, and 58.29 ± 2.67 U/mg protein, *P* < 0.05). These results indicate that PDC can antagonize the reduction of the SOD expression in the human umbilical vein endothelial cell supernatant caused by in vitro cell culture under high glucose conditions.

### 3.5. Effect of PDC on the Level of NO in Cultured HUVEC Cell Culture Supernatant In Vitro

The results are shown in Figure 3(b). Compared with the control group, the NO secreted by HUVECs in the high glucose group significantly reduced (NO concentrations in the culture medium were 83.90 ± 7.19 and 26.85 ± 1.85 *μ*mol/L, *P* < 0.05). Compared with the high glucose group, the NO concentration in the cell culture fluid of the high glucose + PDC (100, 200, and 400 *μ*g/mL) group significantly increased (the concentration of NO in the culture fluid was 44.58 ± 3.87, 53.73 ± 3.12, and 74.91 ± 6.16 *μ*mol/L, *P* < 0.05).

## 4. Discussion

High glucose can aggravate the damage of IR and diabetic vascular endothelial cells [16]. It has been reported that the vascular endothelium-dependent vasodilation function decreases, blood flow velocity decreases, and the vasodilation function is negatively correlated with blood glucose concentration in normal subjects after glucose load, suggesting that high concentrations of glucose can induce the abnormality function of the vascular endothelial's cell. High glucose can cause vascular endothelial cell insufficiency in a variety of ways. The vascular endothelium is an important target for high glucose damage, and oxidative stress injury is an important factor for vascular endothelial damage [17, 18].

Numerous experiments have confirmed that both chronic and intermittent high glucose can increase oxidative stress and promote endothelial cell apoptosis through excessive production of ROS in the mitochondrial electron transport chain level [16, 19, 20]. These observations suggest that antioxidants can play a beneficial role in preventing endothelial cell damage caused by ROS. In this study, to evaluate the effect of PDC on the free radicals produced by endothelial cells caused by high glucose, we measured the content of SOD in the culture medium by colorimetry and the intracellular ROS level by flow cytometry, which is the most commonly used method of free radical generating ability. PDC is one of the main active ingredients of Dendrobium candidum and has good anti-inflammatory, antioxidant, and other biological activities [10, 21–23]. Different concentrations of PDC have been identified by Chen et al. to incresease the activity of SOD, which could contribute to inhibiting oxidative stress and apoptosis of human brain microvascular endothelial cells [24]. The results of this experimental study found that the intracellular ROS level increased significantly, and the activity of SOD in the culture medium decreased under high glucose culture, confirming once again the role of high glucose in oxidative damage and influencing endothelial cell proliferation during incubation with endothelial cells. The dose-dependent addition inhibited the production of ROS caused by high glucose, increased the activity of SOD, and significantly promoted endothelial cell proliferation. Therefore, it can be proved that PDC has a good active oxygen scavenging effect. This is consistent with the results of previous studies [25–27]. NO is a vasoactive substance synthesized by L-arginine as a substrate under the action of endothelial nitric oxide synthase in vascular endothelial cells. A reduction in NO can lead to an accelerated atherosclerotic process. A number of HUVECs in vitro culture experiments have confirmed that the level of NO decreases with the senescence of endothelial cells. In this experiment, we also observed that the NO level of HUVECs induced by high glucose decreased, which is consistent with the results of Hayashi et al. [28], and the experiment proved that PDC could significantly increase NO levels.

This study confirms that under the high glucose environment of 33.3 mmol/L, the proliferation of human umbilical vein endothelial cells is suppressed, and the intracellular calcium concentration and the production of ROS increase, while the activity of SOD and NO production was reduced, but PDC can effectively antagonize the above changes in a dose-dependent manner. PDC can effectively antagonize the damage of HUVECs by high glucose culture, increase the activity of superoxide dismutase, and promote NO release. It is suggested that PDC may be a potential therapy pathway that is very important for protecting vascular endothelial cells. Its protective mechanism may be related to the antioxidant effect of PDC, which provides new ideas for the prevention and treatment of diabetes and vascular complications.

## Figures and Tables

**Table 1:** Effects of different concentrations of Dendrobium candidum polysaccharides (PDC) on the proliferation of HUVEC cells.

Group	OD value
Control group	1.17 ± 0.16
PDC (50 *μ*g/mL)	1.08 ± 0.12
PDC (100 *μ*g/mL)	1.09 ± 0.09
PDC (200 *μ*g/mL)	1.13 ± 0.16
PDC (400 *μ*g/mL)	1.08 ± 0.17
PDC (800 *μ*g/mL)	1.11 ± 0.12

Note: HUVECs were incubated with PDC (50, 100, 200, 400, and 800 *μ*g/mL) for 48 hours. The MTT method was used to detect cell proliferation. All data are expressed as $\bar{x}\pm S$ (*n* = 5).

**Table 2:** Effects of different glucose concentrations on HUVEC cell proliferation.

Group	OD value
Control	1.09 ± 0.14
High glucose (17.5 mmol/L)	1.01 ± 0.12
High glucose (25.5 mmol/L)	0.75 ± 0.09^∗^
High glucose (33.3 mmol/L)	0.42 ± 0.05^∗^
High glucose (40.0 mmol/L)	0.31 ± 0.04^∗∗^
High glucose (56.0 mmol/L)	0.19 ± 0.03^∗∗^
Mannitol (56.0 mmol/L)	1.11 ± 0.13

Note: HUVECs were incubated with D-glucose (17.5, 25.5, 33.3, 40.0, and 56.0 mmol/L) and mannitol (56.0 mmol/L) for 48 hours. The MTT method was used to detect cell proliferation. All data are expressed as $\bar{x\pm S}$ (*n* = 5). ^∗^*P* < 0.05, ^∗∗^*P* < 0.01, compared with the control group.

**Table 3:** Effects of different concentrations of Dendrobium candidum polysaccharides (PDC) on the proliferation of HUVEC cells cultured in high glucose.

Group	OD value
Control	1.29 ± 0.10
High glucose (33.3 mmol/L)	0.39 ± 0.03^∗∗^
High glucose + PDC (100 *μ*g/mL)	0.50 ± 0.05^#^
High glucose + PDC (200 *μ*g/mL)	0.73 ± 0.07^#^
High glucose + PDC (400 *μ*g/mL)	0.89 ± 0.05^##^

Note: both D-glucose (33.3 mmol/L) and PDC (100, 200, and 400 *μ*g/mL) were incubated with HUVECs for 48 hours. The MTT method was used to detect cell viability. All data are expressed as $\bar{x\pm S}$ (*n* = 5). ^∗∗^*P* < 0.05, compared with control group; ^#^*P* < 0.05, ^##^*P* < 0.05 compared with the high glucose group.

## Data Availability

Data generated or analyzed during this study are included in this published article. The datasets generated during and/or analyzed during the current study are available from the corresponding author on reasonable request.
